# A benchmark dataset of herbarium specimen images with label data

**DOI:** 10.3897/BDJ.7.e31817

**Published:** 2019-02-08

**Authors:** Mathias Dillen, Quentin Groom, Simon Chagnoux, Anton Güntsch, Alex Hardisty, Elspeth Haston, Laurence Livermore, Veljo Runnel, Leif Schulman, Luc Willemse, Zhengzhe Wu, Sarah Phillips

**Affiliations:** 1 Meise Botanic Garden, Meise, Belgium Meise Botanic Garden Meise Belgium; 2 Muséum National d’Histoire Naturelle, Paris, France Muséum National d’Histoire Naturelle Paris France; 3 Freie Universität Berlin, Berlin, Germany Freie Universität Berlin Berlin Germany; 4 School of Computer Science & Informatics, Cardiff University, Cardiff, United Kingdom School of Computer Science & Informatics, Cardiff University Cardiff United Kingdom; 5 Royal Botanic Garden Edinburgh, Edinburgh, United Kingdom Royal Botanic Garden Edinburgh Edinburgh United Kingdom; 6 The Natural History Museum, London, United Kingdom The Natural History Museum London United Kingdom; 7 University of Tartu, Tartu, Estonia University of Tartu Tartu Estonia; 8 Finnish Museum of Natural History LUOMUS, Helsinki, Finland Finnish Museum of Natural History LUOMUS Helsinki Finland; 9 Naturalis, Leiden, Netherlands Naturalis Leiden Netherlands; 10 Royal Botanic Gardens Kew, Surrey, United Kingdom Royal Botanic Gardens Kew Surrey United Kingdom

## Abstract

**Background:**

More and more herbaria are digitising their collections. Images of specimens are made available online to facilitate access to them and allow extraction of information from them. Transcription of the data written on specimens is critical for general discoverability and enables incorporation into large aggregated research datasets. Different methods, such as crowdsourcing and artificial intelligence, are being developed to optimise transcription, but herbarium specimens pose difficulties in data extraction for many reasons.

**New information:**

To provide developers of transcription methods with a means of optimisation, we have compiled a benchmark dataset of 1,800 herbarium specimen images with corresponding transcribed data. These images originate from nine different collections and include specimens that reflect the multiple potential obstacles that transcription methods may encounter, such as differences in language, text format (printed or handwritten), specimen age and nomenclatural type status. We are making these specimens available with a Creative Commons Zero licence waiver and with permanent online storage of the data. By doing this, we are minimising the obstacles to the use of these images for transcription training. This benchmark dataset of images may also be used where a defined and documented set of herbarium specimens is needed, such as for the extraction of morphological traits, handwriting recognition and colour analysis of specimens.

## Introduction

Herbarium specimens are a research tool, an archive and a reference for plant sciences. They provide data and verifiability to disciplines such as phytogeography, taxonomy and ecology (Baird 2010). These physical specimens are divided between an estimated 3,000 herbaria worldwide, which makes consultation of all virtually impossible. To facilitate access to the specimens, many herbaria are digitally imaging their collections and making these images available over the internet (Heerlien et al. 2015, Tulig et al. 2012). Although we have a long way to go before full digitisation of the world’s herbaria, there are already about 400 million digitised specimens and the number keeps growing (Thiers 2018). As of November 2018, there are more than 70 million preserved specimen records for plants in the Global Biodiversity Information Facility (GBIF.org 2018).

As digital imaging of the world’s herbaria continues, there is a recognition that large amounts of information can be extracted from these images. This information includes data concerning the specimen's origin on the labels, such as location, date and collector, but also traits and the identity of the plant itself (Corney et al. 2012, MacGillivray et al. 2009, Schuettpelz et al. 2017, Kho et al. 2017). Methods to extract these data are still being developed and require training datasets and test images to validate their effectiveness (Carranza-Rojas et al. 2017).

Herbarium specimens are far from homogeneous. They vary in the language, location and style of the labels, in whether they are typed or handwritten and in the quality and quantity of information on the labels (Mononen et al. 2014). Specimens are frequently annotated by more than one person and are stored by taxon, rather than by collector, both of which make handwriting recognition particularly difficult. A typical specimen will have text written on different dates and by different people in a mixture of printed, typed and handwritten scripts (Vollmar et al. 2010).

Not all herbarium digitisation projects are the same. They vary in aspects, such as the imaging methodology, the resolution of the digital image created and their approach to quality control (Nelson et al. 2012). Anyone building tools to analyse herbarium specimens needs to be aware of these variations and needs to account for them. The language used on the labels can also be problematic. Many collections have specimen labels written in a wide variety of languages, sometimes on the same specimen and one cannot assume the use of Latin script, even in Europe. The interpretation of certain symbols, such as those indicating nomenclatural type status, or of different labels, may not always be clear either.

For all these reasons, we feel it is useful to provide a benchmark dataset of digitised herbarium specimens, made openly available for the development of tools and workflows for data extraction. This dataset has been placed in the public domain specifically to act as a test dataset for research and a benchmark to compare different methods. We have also provided transcribed data, where available, associated with each image, which can be used for comparison or for training systems. In addition, for 250 of the specimens, we have provided image overlays that identify the position of labels. These can be used for segmentation analysis of the specimen.

The images have been released under a Creative Commons Zero licence waiver (https://creativecommons.org/licenses), to ensure that there are no limitations that could hinder or discourage anyone from using them. However, the authors expect users to follow the norms of scientific citation. Each upload of images and data about a specimen has been assigned a DOI (Digital Object Identifier), which will uniquely and persistently identify it to allow citation. Data and media are provided as they were assembled right now and will not be kept in sync with new developments at the collection level after publication. Stable identifiers for the collection specimens themselves can always be found as 'Alternative identifiers' at each upload's landing page (Groom et al. 2017, Güntsch et al. 2017).

## Sampling methods

### Study extent

Curators from nine European herbaria volunteered to provide a sample of their digitally imaged herbarium sheets. Herbarium curators were requested to select specimens following a set of guidelines that were chosen to ensure a representative cross-section of specimen characteristics. The aim was to provide specimens that could answer questions related to the language, condition, age and geography of the specimen and, at the same time, provide a sufficient sample size for statistical analysis (Table 1). Given the different origins and curatorial practices of different collections, not all institutions were capable of following these guidelines in full, particularly if the herbarium did not hold many type specimens (Table 2). For the remainder of this article, we will use the Index Herbariorum codes listed in that table to abrreviate institution names.

### Sampling description

Where possible, images were collected in JPG and lossless TIFF formats. Data were collated as a Darwin Core (DwC) Archive (Darwin Core Task Group, Biodiversity Information Standards (TDWG) 2009), if available from the Global Biodiversity Information Facility (GBIF) using their application programming interface (API) through *rgbif* in the R programming language (Chamberlain 2017, R Core Team 2017). Data were processed in the R language using the *tidyverse* (Wickham 2017) and *rworldmap* (South 2011) packages. Scripts can be found in Suppl. materials 3, 4.

### Quality control

There are no clear indications in any data as to whether a specimen is completely or partially transcribed. The labels may contain a variety of information, so availability of the information in the data is not a good guide to whether it is present on the label. Nevertheless, of the 1,800 specimens, 94% had a collector listed and 56% had either a collector number or an explicit indication that there was no number. A total of 85% had either a verbatim or interpreted date and 90% had either a date or collector number. Hence, most of the specimens have some level of transcription.

**Figure 1.**
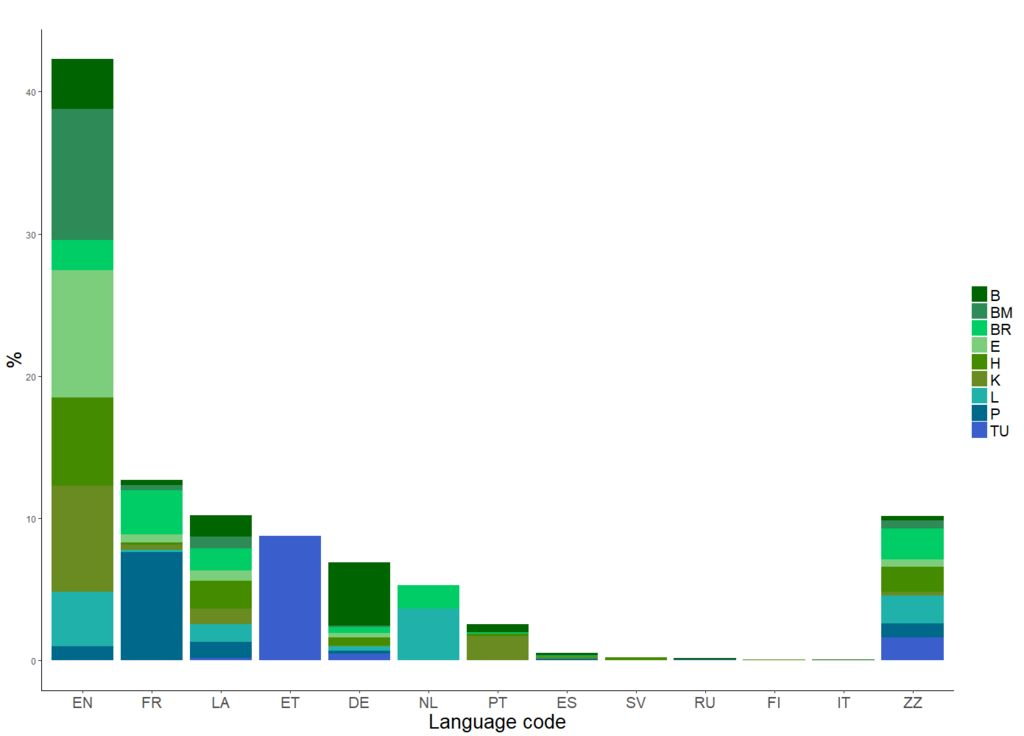
A classification of the languages used on labels of the different specimens. EN = English, FR = French, LA = Latin, ET = Estonian, DE = German, NL = Dutch, PT = Portuguese, ES = Spanish, SV = Swedish, RU = Russian, FI = Finnish and IT = Italian. ZZ indicates a single language could not be determined: either there were multiple languages used on the label, there was no obvious use of a certain language (i.e. only scientific Latin terms) or the language was not readily identifiable. Different herbaria are identified by their Index Herbariorum codes (Institution Code in Table 2).

All specimens were analysed by a polyglot to determine the primary language of the label. In some cases the label had no dominant single language or no other text beyond the scientific Latin name of the specimens. However, the language of the label could be identified for 90% of the dataset (Fig. 1). English appears most frequently, with just over 42%. Five other languages occur in more than 5% (i.e. 90) of specimens: French, Latin, Estonian, German and Dutch. Of these, only Estonian is linked to a single institution. This should make language-based analysis possible with this dataset.

### Step description

As detailed in Table 2, we compiled 200 images from each of nine institutes from seven different countries across Europe. All institutions provided JPEG format images and all but two could also provide TIFF format images; P and L were unable to provide TIFF images due to institution policy. Between institutions, the TIFF file size varied between 25 and 306 MB, with dimensions between 10 and 102 megapixels. Significant differences occurred within institutions too, due to different herbarium sheet sizes as well as different in-house scanning protocols. A total of 150 TIFFs of non-type specimens from H were horizontally photographed in two parts. To reconstruct an image of the whole specimen, these were stitched together in a semi-automated way using Adobe Photoshop CS4. No further image processing of JPEG or TIFF files was done.

For seven of the institutions, the data associated with these images were downloaded using the GBIF API (accessed 2018-07-12). One of the other two (B) provided these data in DwC format directly. The other (H) had no method to export all data in DwC format, so data were extracted in JSON format using their API (https://api.laji.fi) (Accessed 2018-07-09). These data were subsequently mapped to DwC in the R language using the package *jsonlite* (Ooms 2014 and see Suppl. material 2 for the mapping). Data for all specimens were fully joined in the R language. These data are available as Suppl. material 5 as a Comma Separated Value (CSV) file as well as individual JSON-LD files for each image (script for this conversion in Suppl. material 6). Data are provided as they are available now and will not be kept up to date. However, users may be able to download up-to-date data from institutional repositories through the persistent identifiers of the specimens.

For each of a subset of 250 specimens with labels in English, two PNG overlays were manually made in GIMP (GIMP Development Team 2017). These overlays indicate the location and class of labels, stamps, colour charts or other reference objects on the imaged herbarium specimen. One overlay indicates the location of each label with a different colour against a black background (indicated as "_all"). The other overlay indicates the class of labels using a colour code of white for barcode label, yellow for a colour chart and red for any other sort of label (indicated as "_sel"). These overlays can be used to train algorithms to identify labels in order to facilitate data transcription.

## Geographic coverage

### Description

Locations were mapped using their country code and decimal coordinates (Fig. 4). A total of 15% had decimal coordinate values and 94% had a country code. Specimens originate from all continents, except Antarctica.

## Taxonomic coverage

### Description

The higher taxonomy of specimens was determined from the GBIF backbone taxonomy when those data came from GBIF. For the data that did not originate from GBIF, we matched the family (B specimens) or genus (H specimens) in the data to the backbone (GBIF Secretariat 2017). Only seven specimens could not be matched to the backbone. Two had no identification and the other five were homonyms at the genus level (e.g. *Pellaea*, which is both an animal and plant genus). More than 90% of specimens were, not unexpectedly, Tracheophyta, but within this phylum, there was a significant taxonomic coverage of 204 different families in 58 different orders (Fig. 3, Suppl. material 1).

Although we aimed at incorporating 25% nomenclatural type specimens within the dataset, according to their data, only 19% are types. This lower value is because some collections are not created primarily for taxonomy and they therefore do not hold many types. Non-type specimens were selected as specimens without any type status. Hence, some specimens listed as non-types could actually be types, if they had not been identified as such in their digital publication.

Regarding the specimen collector names, there are 1,170 different names associated with the dataset. However, it is likely there are duplicates amongst those 1,170, as some names will not be exact textual matches. Only 6% of the specimens had no collector information.

## Temporal coverage

### Notes

**Figure 2.**
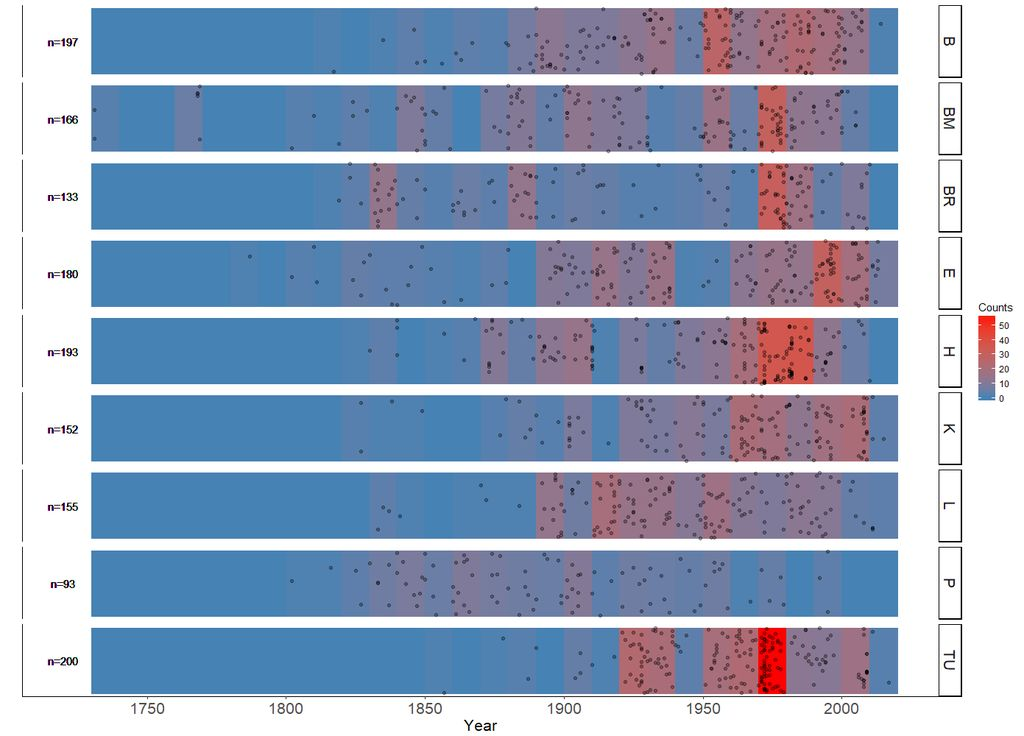
The distribution of collection dates (by year, if known) of the specimens in the dataset for each providing institution. The heat colour indicates the number of specimens for each 10 year time period. Year data were extracted from Darwin Core eventDate and verbatimEventDate if these were in ISO 8601 standard. Codes for the herbaria follow *Table 2*.

**Figure 3.**
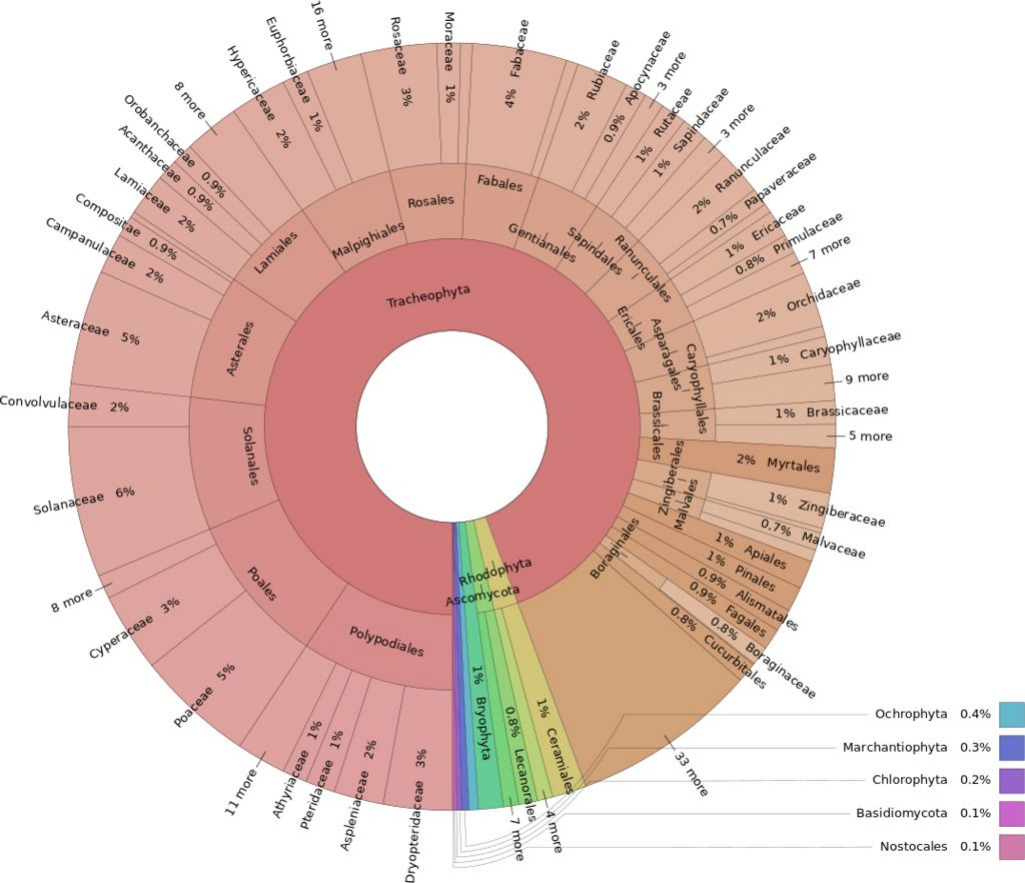
A stacked pie chart generated using Krona (Ondov et al. 2015,  https://github.com/marbl/Krona/), depicting the taxonomic distribution by phylum, order and family (if known). Missing taxa were extracted from the GBIF backbone by family, if possible. For H, they were extracted by genus, as family was unavailable. An interactive version of this graph is available as an HTML file in the supplementary material.

**Figure 4.**
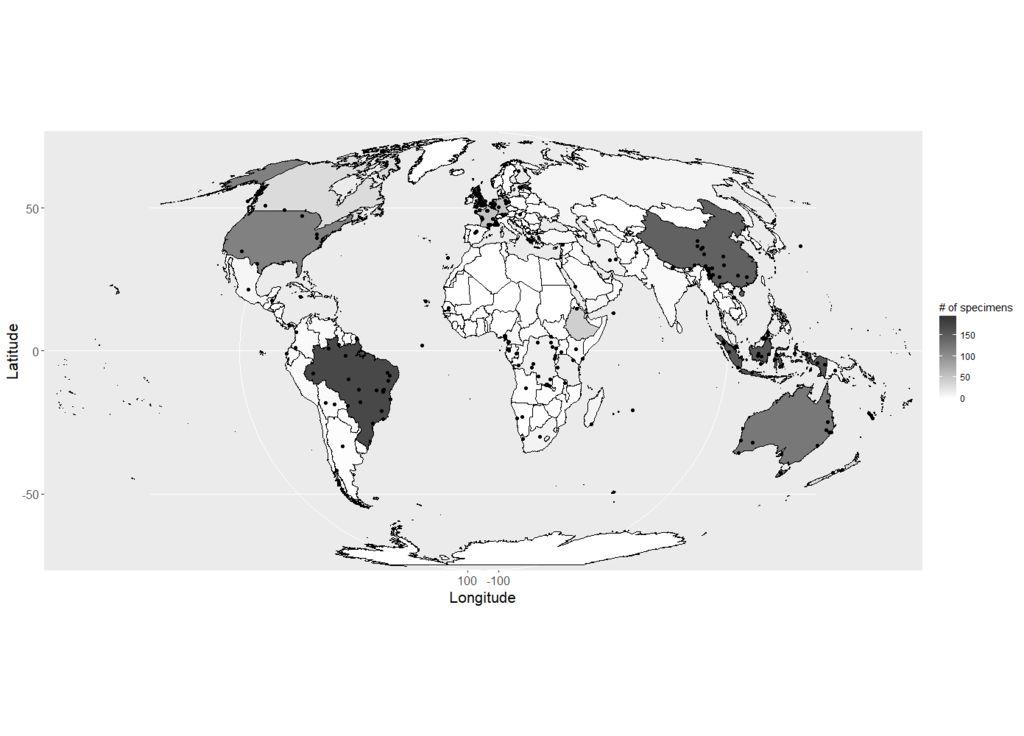
The location of geolocated specimens within the dataset and the number of specimens from each country. A total of 267 (15%) specimens have coordinates associated with them and 1,695 (94%) are located to a country. Both categories may overlap. The map uses a Mollweide equal-area projection.

A broad temporal coverage of the dataset was promoted by forcing a separation at 1970 (Table 1). Year values were derived from the DwC terms eventDate or verbatimEventDate if it was in ISO 8601 format or otherwise standardised (Fig. 2). A year of collection could be identified for 82% of the specimens.

## Usage rights

### Use license

Creative Commons Public Domain Waiver (CC-Zero)

## Data resources

### Data package title

A benchmark dataset of herbarium specimen images with label data: Summary

### Resource link

https://zenodo.org/communities/icedigtest/

### Alternative identifiers

10.5281/zenodo.1492197

### Number of data sets

1

### Data set 1.

#### Data set name

Benchmark Dataset

#### Number of columns

1

#### Description

This landing page contains a CSV file compiling all data associated with herbarium specimens that are part of this dataset, as they could be found on GBIF, JACQ or FinBIF.

In addition, DOIs of the individual specimens uploaded to Zenodo and direct links to the different files (JPEG, TIFF, JSON, PNG) are also included. Index of these added variables:

- persistentID: Persistent Identifier of the collection specimen. The persistent identifier is maintained by each institution and should always lead to the most up-to-date version of a digital specimen record. Apart from the persistent identifier, other data are liable to being amended in institutional databases. Data uploaded as part of this dataset will not be updated with changes at the collection's repository, but this persistent URI will always point to the up-to-date information in the institutional system.

- jpegURL, tiffURL, jsonURL: URLs pointing straight to the respective image and data files themselves, to facilitate (selective) batch downloads.

- pngSegAllURL and pngSegSelURL: Segmented overlays of the herbarium specimens indicating the location of different labels and reference material on the sheet ("All") and their content ("Sel"). More information can be found in the paper (in prep.) associated with this data publication and the individual depositions themselves.

- DOI: The DOI of the deposition of images and data of these specimens on Zenodo. DOIs point to the most up-to-date version of these depositions at the time of the publication of this CSV file. As a rule, this CSV file will be updated should any changes happen to any of the depositions.

- jpegURL2, tiffURL2: A few herbarium sheets had labels on the back and consisted therefore of two scans. As a rule, the label scans are in this category.

**Data set 1.** 

Column label	Column description
Data and links.csv	Supplementary Info 5

## Additional information

As an increasing number of herbarium specimens are digitally imaged, the possibility of automated analysis becomes more attractive. However, simply providing access to the digital images does not enable full use of the resource. The data associated with the image also need to be accessible for most analyses and this requires these data to be digitised, categorised and standardised (Scoble 2010).

The digitisation of label data is one of the most significant bottlenecks to the full digitisation of herbaria (Barber et al. 2013). Digital image capture is only one step towards full digitisation. For this reason, many groups are working on ways to improve and simplify the process of label data capture (Hill et al. 2012, Haston et al. 2015, the ICEDIG Project: http://icedig.eu). Currently, the main method being used is human transcription, either using professional transcribers or volunteers. Professionals may be herbarium employees or outsourcing companies and they may work on bespoke IT systems or online. Volunteers are often recruited online through citizen science portals, such as Notes from Nature (https://www.notesfromnature.org), Les Herbonautes (http://lesherbonautes.mnhn.fr), DoeDat (https://www.doedat.be) and DigiVol (https://digivol.ala.org.au/). These different methods and platforms vary considerably in their approaches to quality control and completeness of transcription. There are many unresolved questions about the success of these different approaches, the quality of the data they generate and their cost-effectiveness (Ellwood et al. 2015). Such questions of quality can be addressed with a benchmark dataset of images, such as the one described in this paper. Trials involving this dataset on the transcription platforms mentioned above are already underway and some had already finished at the time of this article's submission. A publication including a comparatory analysis is planned.

Another approach to data extraction is automation. This might involve optical character recognition of text or other forms of pattern recognition (Drinkwater et al. 2014). Various research groups have had some success in this (Haston et al. 2015). Yet, questions remain on the quality of the data output, including post-processing to classify and standardise the data obtained and how this compares to human transcription. Again, benchmark datasets are required to provide a comparison of techniques. Though, even this diverse set of specimens will not provide training data for every possible need, particularly considering the wide range of languages used on specimens. The subset of 250 image overlays indicating the location and nature of the sheet labels can be used to evaluate the impact of segmenting out the labels before automated data extraction. It can also serve to train algorithms designed to automate label recognition.

Digital images of herbarium specimens may also be used for other purposes, for example, to extract trait data from plants or to identify the species in question (Cope et al. 2012, Carranza-Rojas et al. 2017). However, techniques and software need to be developed to industrialise this to the scale required. It can be expected that techniques of artificial intelligence / machine learning (ML) might be applied for this and the dataset to be used for training and proving purposes (Wäldchen et al. 2018).

Some analysis techniques may only be suitable for certain types of specimen, for example, when ML algorithms are trained only in one language or the handwriting of one collector. Here, we have provided a wide variety of test images from which subsamples can be selected for different purposes. However, in selecting the images, we have not attempted to provide a random subsample of specimens, but have tried to provide a good cross-section of the different kinds. This means that some countries, languages and scripts are not represented at all in the collection and the collection will be biased geographically and taxonomically. However, for those countries and languages represented in the set, there will be multiple specimens.

The whole dataset has been archived to the Zenodo research data repository (https://zenodo.org, Suppl. material 7), where each specimen has its own digital object identifier (DOI). This DOI resolves to a landing page on Zenodo, which contains the specimen’s currently available data as a JSON-LD file and the scanned image in a compressed JPEG format. If available, a lossless TIFF version of the image and two overlay PNG versions can be found there as well. Most data values have also been incorporated into the Zenodo database to improve findability. They are encoded in the "Subjects" fields, combined with persistent identifiers for the Darwin Core and Dublin Core terminology. A landing page with its own DOI for the dataset as a whole contains a CSV file that comprises all available metadata for each specimen and links to the JPEG, PNG and TIFF files. This overarching file should make it easy to download parts of the dataset, such as JPEGs only, TIFFs only or even English specimens only, with simple batch download scripts. The dataset can be viewed at https://zenodo.org/communities/icedigtest. The landing page is available here and its CSV file can also be found in the Supplementary Info (Suppl. material 5).

## Supplementary Material

Supplementary material 1 Taxonomic coverage (interactive HTML file) Data type: Interactive chart
Brief description: Interactive version of the taxonomic coverage chart, Figure 2 in the article. Rendered using Krona (https://github.com/marbl/Krona).
File: oo_224472.html Mathias Dillen

Supplementary material 2 R script used to map data from FinBIF API to DwC Data type: R script
Brief description: This R script was used to obtain metadata for the specimens from H in Darwin Core format, using the FinBIF API. Certain transformations depend on what was present in this specific dataset and might not be generically applicable.
File: oo_224474.R Mathias Dillen

Supplementary material 3 R script CSV files Data type: Zipped CSV files
Brief description: This ZIP contains the CSV files necessary for the R script which retrieved and joined the metadata of the dataset and produced most of the graphs.
In addition to seven files with 200 barcodes each for BR, BM, E, K, L, P and TU and two files containing all metadata for B and H, it also contains a file listing the label language for each specimen, a summary table for languages in the dataset and a file mapping DwC terms to their overarching categories.
File: oo_224475.zip Mathias Dillen

Supplementary material 4 R script used for this paper Data type: R script
Brief description: This R script file contains the different scripts used to obtain metadata, join it, export it and produce the paper's graphs (except for the taxonomic graph, which was done using data exported from R into the Krona Excel macro template, which can be found on Github). The CSV files needed for this script are in a separate ZIP file.
File: oo_224646.R Mathias Dillen

Supplementary material 5 Table of specimen data, DOIs and URIs Data type: CSV
Brief description: This file contains data of the 1800 digitised specimens this paper's dataset is composed of. The joined data originate from different sources as described above and have also been filtered for a few repository-specific variables, such as GBIF taxon keys. DwC extensions are encoded in JSON.
This file also contains a list of DOIs and Zenodo file URIs (jpegURI, tiffURI...) for the images of each specimen this dataset consists of. Using these links and DOIs, it should be easy to retrieve and cite any proportion of this dataset as needed.
File: oo_244128.csv Mathias Dillen

Supplementary material 6 R Script to compile JSON files from CSV Data type: R script
Brief description: This R script was used to convert data in a CSV format to single JSON-LD files. The ZIP file also contains the original CSV file.
File: oo_230010.zip Mathias Dillen

Supplementary material 7 Python script to upload the dataset to Zenodo Data type: Python Script
Brief description: This Python script was used to upload the dataset to the Zenodo platform through their API.
File: oo_241268.py Mathias Dillen

## Figures and Tables

**Table 1.** The guidelines given to herbaria to select specimens for the test dataset. The goal was not to have a representative sample of all specimens, but to have comparable subsets, which will have labels written in different languages; will be printed or handwritten; will cover a wide range of dates; will be both type specimens and general collections and will provide specimens from different families and different parts of the world.

Number of specimens	Type status	Date of collection	Geography
25	Type	< 1970	Any country
25	Type	> 1970	Any country
25	non-Type	< 1970	From the country where the herbarium is located
25	non-Type	> 1970	From the country where the herbarium is located
100	non-Type	Any	non-Type specimens from one other country or region of which the herbarium possesses a substantial number of specimens

**Table 2.** Contributions of 9 different institutes to the dataset. Availability of JPG and TIFF images is indicated, as well as the source of label data. Most institutes were able to follow the template in Table 1. The regions picked for the 100 non-type specimens are indicated in the last column, as are deviations from the template in Table 1. Institution codes follow Index Herbariorum (http://sweetgum.nybg.org/science/ih/). The DOI of the collections is listed if GBIF was used as a data source. FinBIF is the Finnish Biodiversity Information Facility available at www.species.fi (Schulman et al. 2018). JACQ is a joint specimen data management system of over 30 European and Asian herbaria available at https://herbarium.univie.ac.at/database/ (Rainer and Vitek 2009).

**Institute**	**Institution Code**	**Data Source**	**Composition (with ISO 3166-1 alpha-2 Country Codes)**
**Meise** Botanic Garden	BR	10.15468/wrthhx	As Table 1; 100 from AU, CA, NZ, US
Royal Botanic Gardens, **Kew**	K	10.15468/ly60bx	As Table 1; 100 from BR
Natural History Museum, **London**	BM	10.5519/0002965	As Table 1; 100 from AU, CA, NZ, US
Botanic Garden and Botanical Museum, **Berlin**	B	JACQ	As Table 1; 100 from AU, BR, CN, ID, TZ, US
Royal Botanic Garden **Edinburgh**	E	10.15468/ypoair	As Table 1; 100 from CN
National Museum of Natural History, **Paris**	P	10.15468/nc6rxy	50 type, 50 non-Type FR, 100 non-Type not FR
Natural History Museum, University of **Tartu**	TU	10.15156/bio/587444	100 < 1970, 100 > 1970
**Naturalis** Biodiversity Center	L	10.15468/ib5ypt	As Table 1; 100 from ID; no selection on date
Finnish Museum of Natural History LUOMUS, University of **Helsinki**	H	FinBIF	As Table 1; 14 FI, 36 ET instead of 50 FI; 100 from AU, BR, CN, ID, US
