# Supplementary material for: A benchmark dataset of herbarium specimen images with label data
Source: Biodivers Data J. 2019 Feb 8;(7):e31817. doi: 10.3897/BDJ.7.e31817 (PMC6396854; doi:10.3897/BDJ.7.e31817)
Supplement: Supplementary material 1 — Taxonomic coverage (interactive HTML file) [file bdj-07-e31817-s001.html]

Javascript must be enabled to view this page.

magnitude

 2012

 29

 3

 1

 2

 16

 1

 3

 2

 1

 8

 1

 1

 1

 1

 1

 3

 2

 1

 1

 1

 3

 1

 2

 1

 1

 2

 1

 1

 1

 1

 21

 6

 2

 3

 1

 1

 1

 6

 3

 1

 1

 1

 2

 1

 1

 1

 1

 5

 3

 2

 4

 1

 1

 2

 1

 1

 1

 1

 2

 2

 1

 1

 6

 3

 1

 1

 1

 3

 1

 1

 1

 8

 1

 1

 4

 4

 1

 1

 2

 2

 42

 3

 3

 29

 5

 4

 1

 3

 14

 2

 8

 1

 1

 1

 1

 4

 1

 1

 1

 1

 1898

 19

 1

 6

 3

 6

 3

 24

 14

 6

 3

 1

 1

 1

 3

 2

 1

 56

 1

 3

 1

 1

 3

 47

 154

 1

 97

 34

 18

 1

 3

 17

 16

 1

 40

 20

 2

 1

 14

 1

 2

 1

 1

 54

 2

 10

 2

 24

 1

 1

 1

 1

 11

 1

 5

 5

 3

 3

 3

 1

 1

 1

 17

 10

 7

 4

 3

 1

 2

 2

 8

 7

 1

 10

 1

 9

 2

 2

 58

 4

 1

 23

 1

 2

 16

 6

 1

 4

 90

 80

 5

 5

 18

 7

 1

 7

 1

 2

 79

 18

 2

 9

 6

 44

 4

 4

 2

 2

 1

 1

 2

 2

 2

 2

 119

 18

 2

 10

 36

 4

 18

 2

 11

 8

 1

 9

 14

 7

 7

 8

 2

 4

 2

 8

 8

 12

 10

 2

 117

 1

 1

 4

 2

 1

 1

 22

 2

 48

 3

 2

 3

 2

 4

 3

 11

 1

 6

 25

 1

 2

 15

 2

 5

 38

 1

 8

 14

 13

 2

 2

 2

 2

 2

 2

 2

 7

 1

 2

 2

 2

 1

 1

 21

 5

 1

 6

 3

 1

 5

 10

 1

 9

 187

 1

 67

 3

 4

 3

 1

 100

 1

 2

 5

 188

 42

 21

 3

 5

 1

 3

 1

 58

 1

 1

 2

 10

 25

 10

 5

 6

 6

 61

 6

 2

 15

 38

 94

 2

 20

 2

 64

 2

 4

 2

 2

 6

 6

 66

 8

 2

 12

 22

 22

 14

 8

 2

 4

 4

 2

 2

 1

 1

 164

 36

 128

 6

 6

 34

 2

 2

 30
